# Comparative effectiveness of peroneus longus tendon (PLT) autografts versus hamstring tendon (HT) autografts in anterior cruciate ligament reconstruction: a comprehensive systematic review and meta analysis

**DOI:** 10.1007/s00590-024-03984-w

**Published:** 2024-05-16

**Authors:** Jae Yong Park, André Fernandes, Shin Young Park, Hayeon Lim, Iqbal Farhan Sayudo, Liron Leibovitch, Elcio Machinski, Joon Ha

**Affiliations:** 1 Department of Medicine, Imperial College London, Ayrton Rd, South Kensington, London, SW7 5NH UK; 2 Department of Trauma and Orthopaedics, York and Scarborough NHS Trust, London, UK; 3 Department of Medicine, Manchester University, Manchester, UK; 4 Department of Medicine, Syiah Kuala University, Banda Aceh, Indonesia; 5 Department of Medicine, Bar-Ilan University, Safed, Israel; 6 Department of Medicine, State University of Ponta Grossa, Ponta Grossa, Brazil; 7 St Michael’s Hospital, University of Toronto, Toronto, Canada

**Keywords:** Peroneus longus tendon, Hamstring tendon, Autograft, Anterior cruciate ligament, Reconstruction

## Abstract

The hamstring tendon (HT) autograft is currently the most widely utilised autograft option for anterior cruciate ligament (ACL) reconstruction. However, recent studies endorse the peroneus longus tendon (PLT) autograft as a viable alternative. To evaluate this, we systematically reviewed randomised controlled trials (RCTs) to compare the efficacy of PLT against HT autografts. Our search encompassed Cochrane, Embase, OVID, PubMed, and Scopus databases for RCTs comparing outcomes of PLT and HT autografts in ACL reconstruction. Primary outcomes included Lysholm and International Knee Documentation Committee (IKDC) scores, while secondary outcomes involved American Orthopaedic Foot and Ankle Society (AOFAS) scores, graft diameters and donor-site complications. Statistical analysis was performed using Review Manager 5.4 (Cochrane Collaboration) and heterogeneity was assessed with I^2^ statistics. 683 patients from 6 RCTs were included, with 338 (49.5%) patients treated with PLT autografts. Follow-up ranged from 12 to 30 months. Despite lower preoperative Lysholm scores in the PLT group, no significant differences were observed at 6 and 12 months. Although preoperative and 6-month IKDC scores were lower in the PLT group, no significant differences were found at 12 and 24 months. AOFAS scores showed no significant preoperative difference, but slightly lower scores were noted in the PLT group at 12 or 24 months. There was no significant difference in graft diameter, while donor-site complications were fewer in the PLT group. In summary, the PLT autograft is a promising and non-inferior alternative to the HT autograft, demonstrating equivalent outcomes in patient-reported knee and ankle metrics, comparable graft diameters and fewer donor-site complications.

## Introduction

Anterior cruciate ligament (ACL) reconstruction is considered the gold standard treatment for ACL rupture, especially in young, highly active individuals or those with significant anterior tibial subluxation and intra-articular damage [1, 2]. This procedure generally involves arthroscopically removing the damaged ACL and replacing it with a graft. There are a diverse range of graft types including autografts, allografts, synthetic, xenografts, and hybrid grafts.

The hamstring tendon (HT) autograft currently stands as the most commonly used autograft for ACL reconstruction [3]. Since its inception in 1934 by Galeazzi [4], the use of HT autografts has undergone several modifications, including variations in distal graft attachment, fixation methods and the number of strands (2, single bundle; 4, double bundle) [5]. Despite the various modifications, the underlying principle remains consistent—either the semitendinosus and/or gracilis tendons are harvested at their proximal insertion [5]. The HT autograft is thought to be the preferred choice due to its many advantages including reduced postoperative pain and thigh muscle atrophy [6]. However, it is not without its drawbacks, such as graft size variability and challenges in graft fixation [6]. There are also other commonly used alternatives like the patellar tendon (PT) or quadriceps tendon (QT) autografts, each with their own set of pros and cons.

Another alternative that has shown significant promise is the peroneus longus tendon (PLT) autograft. Despite not being commonly used in practice, recent studies have shown that PLT autografts may produce comparable graft diameters, outcomes, and donor site morbidity to its counterparts [7–9]. Exploring the effectiveness of the PLT autograft has the potential to widen the range of available graft options for ACL reconstruction. This is important as choosing a graft is a patient-centric decision that requires considering the patient’s unique anatomy, preferences, and needs. Therefore, our primary aim is to address the viability of the PLT autograft as a non-inferior alternative to the conventional HT autograft via a systematic review and meta-analysis (SRMA).

While previous SRMAs have evaluated the comparison between PLT and HT autografts, their limitations stem from drawing conclusions from studies with a broad level of evidence range (1–4). Therefore, our updated SRMA aims to distinguish itself by solely investigating level 1 randomised controlled trial (RCT) studies.

## Methods

The SRMA was conducted and reported in accordance with the guidelines outlined in the Cochrane Collaboration Handbook for Systematic Review of Interventions and the Preferred Reporting Items for Systematic Reviews and Meta-Analysis (PRISMA) Statement [10].

### Search strategy

A comprehensive systematic search was performed on Cochrane, EMBASE, OVID, PubMed, and Scopus databases from inception to December 7, 2023. The search strategy consisted of the term “peroneus longus” or “fibularis longus” and at least one of the following terms: acl, “anterior cruciate ligament”, “acl reconstruction”, or “anterior cruciate ligament reconstruction”.

### Eligibility criteria and study selection

This SRMA focused on incorporating studies that satisfied the following eligibility criteria: (1) RCTs; (2) comparing PLT to HT autografts; (3) in patients undergoing primary ACL reconstruction surgery; (4) with a follow-up of at least 1 year; (5) reporting any outcome of interest. The exclusion criteria encompassed: (1) studies involving participants aged < 15 years; (2) those undergoing revision ACL surgery or other types of knee ligament surgery; (3) the utilisation of allografts or hybrid grafts; (4) articles not in English; and (5) other article types, including animal studies, biomechanical studies, cadaveric studies, case reports, technical notes, conference abstracts, comments, editorials, and letters. In order to identify further relevant studies beyond the search strategy, a manual examination of the references from the included studies as well as prior systematic reviews that align with the scope of our research was carried out. Two independent reviewers (J.Y.P., A.F.) autonomously conducted the literature search according to predetermined criteria, performed quality assessments, and collaboratively reviewed the included articles to extract relevant data. The prospective SRMA protocol was officially registered on PROSPERO with the identifier #CRD42023482367 on November 14, 2023.

### Data extraction

The authors gathered the following information from the studies: (1) baseline characteristics of the included studies; (2) primary outcomes, involving Lysholm and International Knee Documentation Committee (IKDC) scores before and after surgery at 6, 12, and 24 months; and (3) secondary outcomes, including American Orthopaedic Foot and Ankle Society (AOFAS) scores before and after surgery at 6, 12, and 24 months, as well as graft diameters and donor-site complications.

### Quality assessment

The evaluation of bias risk was independently carried out by two authors (J.Y.P., S.Y.P.). Both authors employed the Risk of Bias tool for Randomised Trials (ROB-2) in accordance with Cochrane’s recommendations [11]. In cases of discrepancies, resolution was achieved through discussion involving the senior author (J.H.), addressing the underlying reasons for any disagreements.

### Data analysis

Continuous outcomes were compared through mean differences (MD), and binary endpoints were evaluated using risk ratios (RR), each accompanied by their respective 95% confidence intervals (CI). The DerSimonian and Laird method was employed in order to conduct the meta-analysis with random effects. In cases of missing mean and standard deviation (SD), calculations were derived from the median, minimum, and maximum, following the approach outlined by Luo et al. [12]. The statistical analyses were carried out using Review Manager 5.4 (The Cochrane Collaboration, London, UK).

### Assessment of heterogeneity

The Cochrane’s Q test and I^2^ statistics were used to assess heterogeneity. Consequently, a significance in heterogeneity was determined by *p* values below 0.10 and I^2^ values exceeding 25%. The interpretation of the heterogeneity measures adhered to the guidelines provided in the Cochrane Handbook for Systematic Reviews of Interventions.

## Results

### Literature search

**Fig. 1:**
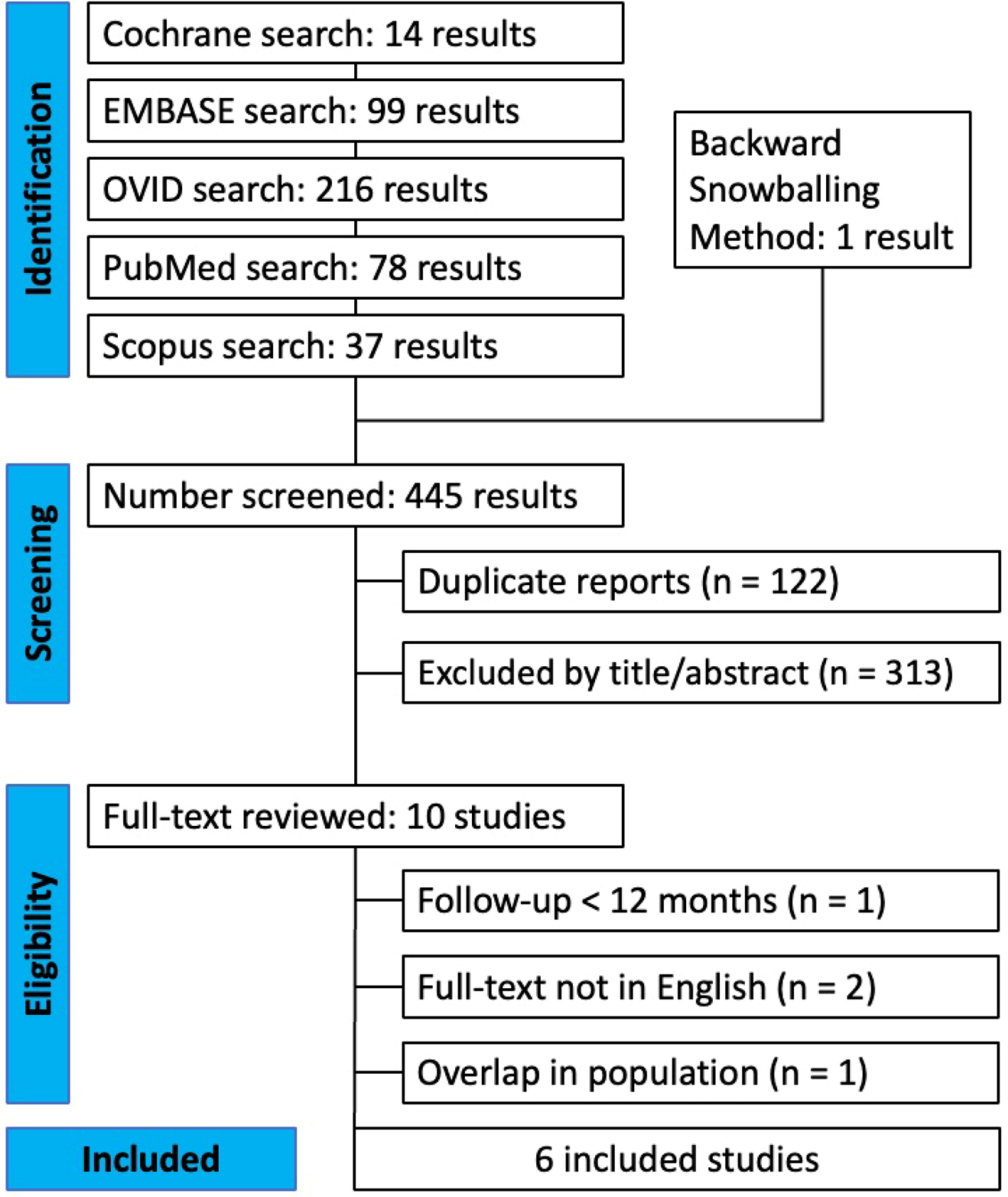
PRISMA flow diagram of study screening and selection

As illustrated in Fig. 1, the literature search produced 444 articles from Cochrane (n = 14), EMBASE (n = 99), OVID (n = 216), PubMed (n = 78), and Scopus (n = 37). One article was discovered upon reviewing the reference lists of the included articles or otherwise known as the ‘backward snowballing’ method. After the removal of 122 duplicate entries, two independent reviewers (J.Y.P., A.F.) screened the remaining articles, excluding 313 articles by title/abstract. This left 10 papers for a thorough review of the full-text, of which, 4 were excluded as they did not satisfy the inclusion criteria. Any discrepancies were discussed and resolved between the first (J.Y.P.) and senior author (J.H.). Ultimately, a total of 6 RCTs were included in this SRMA [13–18].

### Characteristics of the included studies and quality assessment

The baseline characteristics of the included studies are reported in Table 1. The analysis included a total of 683 patients from 6 RCTs comparing PLT and HT autografts for ACL reconstruction surgery. Among them, 338 (49.5%) patients were treated with PLT autografts, and 345 (50.5%) were treated with HT autografts. Follow-up durations ranged from 12 to 30 months.

**Table 1:** Baseline characteristics of included RCT studies [13–18]

Study	Journal	Time period	Autograft type		Patients, n (%)		Male, n (%)		Age (years)		Follow-up (months)
			PLT	HT	PLT	HT	PLT	HT	PLT	HT	
Agarwal [13]	Cureus	2020–2021	Tripled	Quadrupled	98 (50.5)	96 (49.5)	68 (69.4)	57 (59.4)	28.00 ± 4.91	27.5 ± 4.06	12
Bi [14]	The Journal of Knee Surgery	2013–2015	Quadrupled AH	Quadrupled	62 (50.0)	62 (50.0)	34 (54.8)	31 (50.0)	29.1 ± 6.5	27.9 ± 6.7	30
Saeed [15]	The Journal of Bone and Joint Surgery	2017–2021	Doubled	Quadrupled	121 (52.2)	111 (47.8)	NR	NR	NR	NR	24
Shi [16]	The Journal of Knee Surgery	2002–2012	Doubled	Quadrupled	18 (47.4)	20 (52.6)	NR	NR	40.9 ± 10.70^a^	39.26 ± 8.83^a^	24
Vijay [17]	Journal of Orthopaedics,Trauma, and Rehabilitation	2019–2020	Doubled	Quadrupled	23 (51.1)	22 (48.9)	17 (73.9	18 (81.8)	33.57 ± 9.54	31.82 ± 9.62	12
Waly [18]	The Egyptian Orthopaedic Journal	2019–2020	NR	NR	25 (50.0)	25 (50.0)	19 (76.0)	17 (68.0)	33.3 ± 6.4	31.5 ± 3.9	12

*AH* anterior half, *HT* hamstring tendon, *NR* not reported, *PLT* peroneus longus tendon

^a^Estimated mean ± standard deviation obtained from median, minimum and maximum values via the method by Luo et al. [12] as recommended by the Cochrane Collaboration

The risk of bias analysis utilised the ROB-2 tool for randomised trials, following Cochrane's recommendations [11]. Among the included studies, 5 exhibited some concerns regarding the overall risk of bias, predominantly in 2–3 domains. Notably, only one study demonstrated an overall low risk of bias. A comprehensive summary of the risk of bias analysis is presented in Table 2.

**Table 2:** Risk of bias for included RCT studies [13–18]

Study	Bias from randomization process	Bias due to deviations from intended interventions	Bias due to missing outcome data	Bias in measurement of the outcomes	Bias in selection of the reported result	Overall risk of bias
Agarwal [13]	Some concerns	Some concerns	Low risk	Some concerns	Low risk	Some concerns
Bi [14]	Low risk	Low risk	Low risk	Low risk	Low risk	Low risk
Saeed [15]	Low risk	Some concerns	Low risk	Some concerns	Low risk	Some concerns
Shi [16]	Low risk	Some concerns	Low risk	Some concerns	Low risk	Some concerns
Vijay [17]	Low risk	Some concerns	Low risk	Some concerns	Low risk	Some concerns
Waly [18]	Some concerns	Some concerns	Low risk	Some concerns	Low risk	Some concerns

Given the exclusive focus on RCTs in this SRMA, and with the lack of patients switching treatments from their initially randomised allocation, an intention-to-treat analysis approach was employed. This allowed for the prognostic balance of randomisation to be maintained.

### Results from the study

**Fig. 2:**
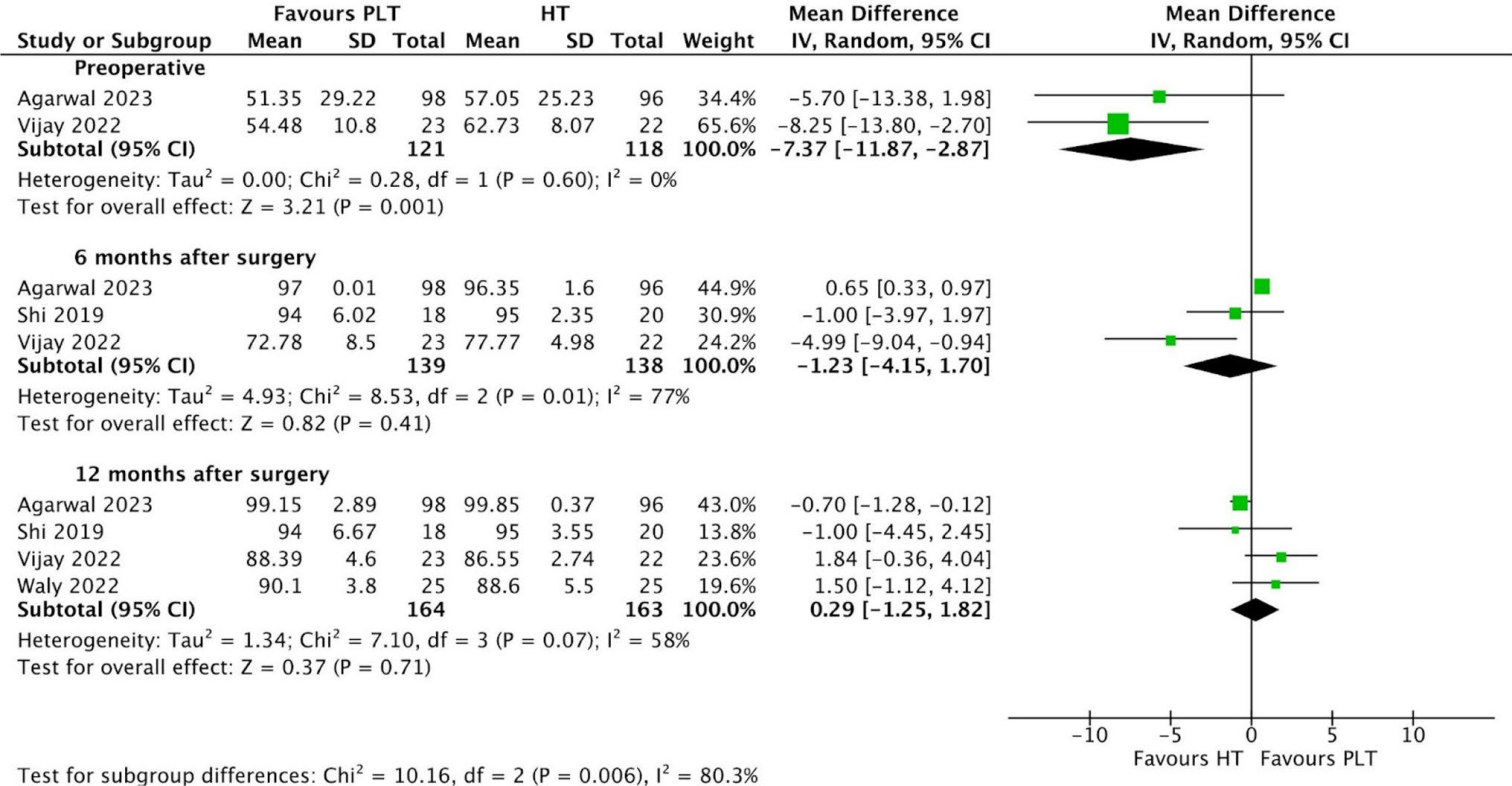
Comparison of lysholm scores over time: preoperative, 6, and 12 months after surgery

Patients undergoing ACL reconstruction surgery with PLT autografts exhibited lower preoperative Lysholm scores (MD − 7.37; 95% CI − 11.87 to − 2.87; *p* = 0.001; I^2^ = 0%) (Fig. 2) compared to those with HT autografts. This difference was statistically significant, as the *p* value was less than 0.05 and the 95% CI did not include 0, the value of no difference. The PLT group also exhibited lower Lysholm scores at 6 months (MD − 1.23; 95% CI − 4.15 to 1.70; *p* = 0.41; I^2^ = 77%), however these differences were not statistically significant. Conversely, at 12 months after surgery (MD 0.29; 95% CI − 1.25 to 1.82; *p* = 0.71; I^2^ = 58%), the Lysholm scores were slightly higher in the PLT group than in the HT group. Nonetheless, these differences were also not statistically significant.

**Fig. 3:**
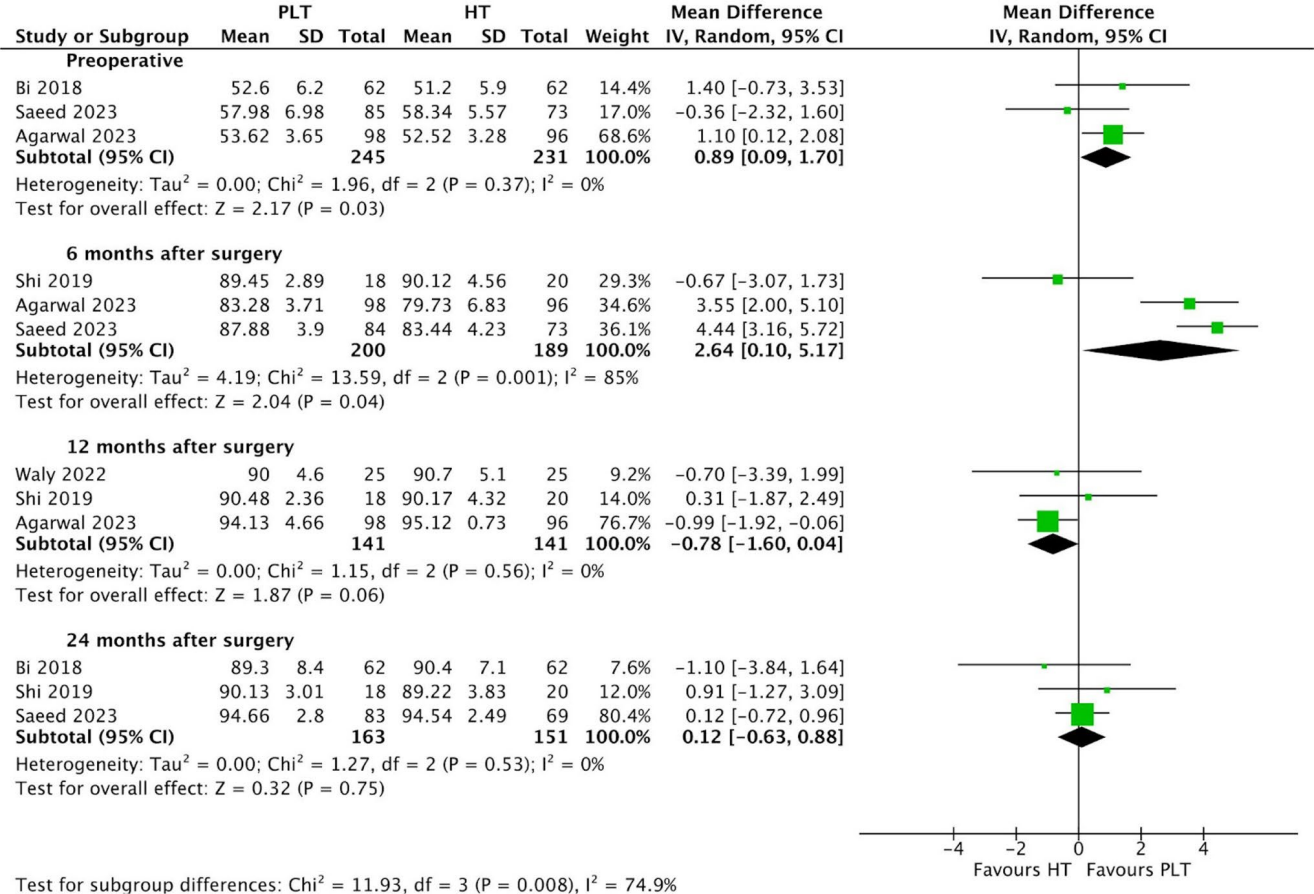
Comparison of IKDC scores over time: preoperative, 6, 12, and 24 months after surgery

Patients in the PLT autograft group demonstrated slightly higher preoperative IKDC scores (MD 0.89; 95% CI 0.09–1.70; *p* = 0.03; I^2^ = 0%) (Fig. 3) compared to those in the HT autograft group. The PLT autograft group also exhibited larger IKDC scores 6 months after surgery (MD 2.64; 95% CI 0.10–5.17; *p* = 0.04; I^2^ = 85%). These differences in IKDC scores preoperatively and at 6 months after surgery were statistically significant. On the other hand, at 12 months after surgery, patients treated with PLT autografts had slightly lower IKDC scores (MD − 0.78; 95% CI − 1.60 to 0.04; *p* = 0.06; I^2^ = 0%) compared to those treated with HT autografts. However, this difference was not statistically significant. Additionally, at 24 months after surgery, IKDC scores were once again higher in the PLT autograft group (MD 0.12; 95% CI − 0.63 to 0.88; *p* = 0.75; I^2^ = 0%) compared to the HT autograft group, yet these differences were also not statistically significant.

**Fig. 4:**
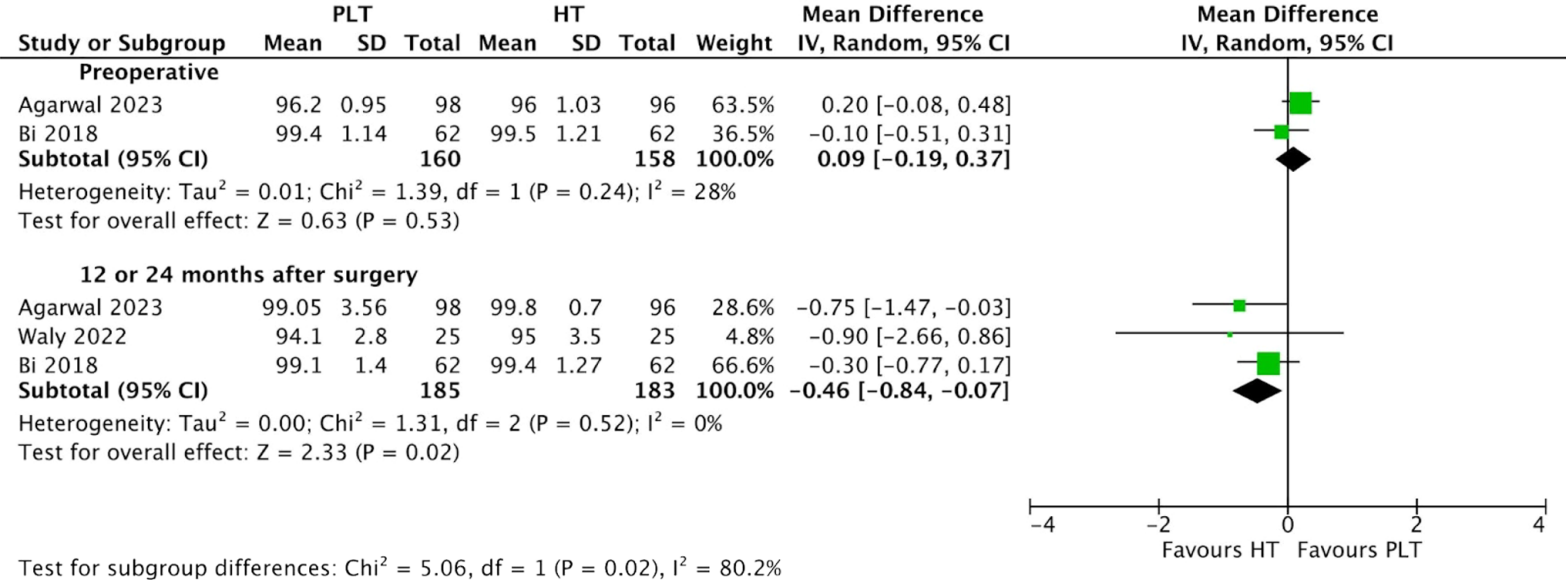
Comparison of AOFAS scores over time: preoperative and 12 or 24 months after surgery

Patients undergoing ACL reconstruction surgery with PLT autografts showed slightly higher preoperative AOFAS scores (MD 0.09; 95% CI − 0.19 to 0.37; *p* = 0.53; I^2^ = 28%) (Fig. 4) compared to those with HT autografts; however, this difference was not statistically significant. Conversely, AOFAS scores were slightly lower in the PLT autograft group (MD − 0.46; 95% CI − 0.84 to − 0.07; *p* = 0.02; I^2^ = 0%) at 12 or 24 months than the HT autograft group. This difference was found to be statistically significant.

**Fig. 5:**
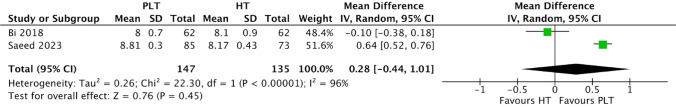
Comparison of graft diameters

The diameter of the PLT autografts was larger (MD 0.28; 95% CI − 0.44 to 1.01; *p* = 0.45; I^2^ = 96%) (Fig. 5) than the HT autografts. Nevertheless, this difference did not reach statistical significance, as indicated by the *p* value exceeding 0.05 and the 95% CI including 0.

**Fig. 6:**
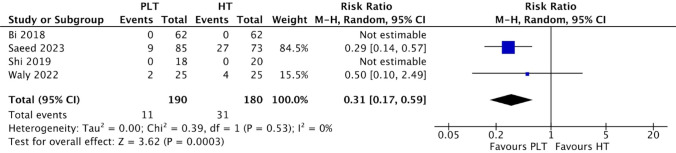
Comparison of donor-site complications

The PLT group exhibited a lower incidence of donor-site complications (RR 0.31; 95% CI 0.17–0.59; *p* = 0.0003; I^2^ = 0%) (Fig. 6) in comparison to the HT group. This difference achieved statistical significance, as indicated by a *p* value below 0.05 and a 95% CI that did not include 1.

## Discussion

In this SRMA, we included a total of 6 RCTs involving 683 patients. These studies analysed homogenous data and documented patient-reported outcomes using Lysholm, IKDC and AOFAS scores, graft diameters, and total complications comparing the use of PLT autografts with HT autografts for ACL reconstruction surgery. The main findings are: (1) lower preoperative Lysholm sores in the PLT group, but no significant differences in Lysholm scores between the groups 6 and 12-months after surgery; (2) higher preoperative and 6-month IKDC scores in the PLT group, but no significant differences in the 12 and 24-month IKDC scores between the groups; (3) no significant differences in preoperative AOFAS scores between the groups, but lower 12 or 24-month AOFAS scores in the PLT group; (4) No significant differences in graft diameters between the groups; (5) Lower incidence of donor-site complications in the PLT group.

However, it is important to note that statistical significance does not always translate to practical clinical significance. The first set of statistically significant findings was the lower preoperative Lysholm scores in the PLT group (MD − 7.37). Considering that the minimal clinically important difference (MCID) for the Lysholm score has been reported to be 10 [19], this suggests that the observed difference is not likely to be clinically significant. The second set of statistically significant findings was the higher preoperative (MD 0.89) and 6-month (MD 2.64) IKDC scores in the PLT group. Given the subjective nature of the IKDC score, and its reported MCID of 11–20 [19], these small differences in IKDC scores most likely lack clinical significance. However, it’s important to note that since preoperative scores serve as a baseline characteristic, there technically should not be any meaningful differences between the groups. Consequently, these statistically significant disparities in preoperative Lysholm and IKDC scores suggest a potential presence of selection and/or confounding bias. The third set of statistically significant findings was the lower 12 or 24-month AOFAS scores (MD − 0.46) in the PLT group. Like the IKDC score, the AOFAS score is also a subjective assessment on a scale of 100 with a reported MCID of 7.9–30.2 [20], therefore a difference of − 0.46 is unlikely to hold clinical significance. The final set of statistically significant findings was the lower incidence of donor-site complications (RR 0.31) observed in the PLT group. This implies that individuals treated with PLT autografts are 0.31 times less likely to develop donor-site complications than those treated with HT autografts, representing a clinically significant statistic. However, it’s crucial to acknowledge that two out of the four studies reporting complications recorded zero incidents in both PLT and HT groups, rendering the RR not estimable. Consequently, the calculation of the average RR consisted of only two studies. So while the RR may be statistically significant, its limited generalisability underscores the need for more studies to obtain a more clinically representative and accurate statistic.

There are other limitations to our study. Our study was limited by a small overall sample size, due to the limited number of RCTs on the topic. This is most likely attributed to the low volume of ACL reconstruction surgeries with the PLT autograft. Therefore, more RCTs comparing PLT and HT autografts should be carried out in order for more representative conclusions to be made. Our study was also limited by its short follow-up period. While the PLT autograft appears to be a solid alternative for HT autograft based on 6, 12 and 24 month outcomes, there is no certainty that this conclusion can be extended to longer follow-up periods, such as for 5 years after surgery. Therefore, RCTs with longer follow-up periods should be carried out for a more comprehensive understanding of the long-term outcomes, durability, and complications associated with ACL reconstruction with PLT autografts in relation to HT autografts. In addition to this, according to the ROB-2 tool for randomised trials, only 1 RCT presented a “low risk of bias” while the other 5 studies presented “some concerns”. Although this SRMA only focuses on level 1 RCTs, the prevalence of studies with “some concerns” regarding bias may negatively impact the reliability of the results. Finally, the results for the Lysholm scores at 6 (I^2^ = 77%) and 12 months (I^2^ = 58%) after surgery, IKDC scores at 6 months (I^2^ = 85%) after surgery, preoperative AFOAS scores (I^2^ = 28%) and graft diameter (I^2^ = 92%) were all significant for heterogenity. This is most likely due to a wide variety of variables, including selection and confounding bias, as mentioned within the studies themselves [13–18]. In order to mitigate these sources of heterogeneities for more accurate comparisons, future studies should aim to incorporate more robust study methodologies, minimise potential biases, and establish more standardised protocols for outcome measurement.

Despite these limitations, there are multiple other studies which corroborate our findings. Quinn et al. conducted a systematic review of level 1–4 studies, indicating that PLT autografts can generate grafts of adequate size with early outcomes akin to HT autografts and a diminished risk of donor site morbidity [21]. However, this study was also limited by a short follow-up duration, significant heterogeneity, and insufficient research demonstrating supplementary advantages of PLT autografts, especially concerning morbidity outcomes [21].

Lower postoperative complications were also identified in a SRMA by Shi et al. [22]. This review compared PLT and HT autografts from controlled trials sourced from both Chinese and English databases [22]. It was revealed that there were no significant differences in postoperative Lysholm and IKDC scores at 6 and 12 months. Moreover, there were no significant differences between both groups in Tegner scores, degree of joint motion, and degree of joint relaxation at 6 and 12 months post-surgery.

A RCT conducted by Zuo et al., comprising 82 elderly subjects undergoing ACL reconstruction with either PLT or HT autografts reaffirms the PLT as a non-inferior option for ACL rupture management [23]. Their study demonstrated larger IKDC and Lysholm scores in the PLT group at both 6 and 12 months post-surgery, and this was coupled with an enhanced range of motion (ROM) compared to the HT group at the 12-month postoperative assessment [23]. Furthermore, a greater proportion of patients in the PLT group exhibited minimal to no laxity in the anterior drawer tests at 12 months after surgery compared to their counterparts in the HT group [23].

Another RCT by Shair et al., encompassing 80 ACL rupture cases with a brief 9-month follow-up, advocated for the utilisation of PLT as a preferred autograft option [24]. The investigation underscored reduced incidences of donor-site related morbidities, notably among individuals engaged in frequent kneeling during daily activities [24].

The suitability of PLT as an autograft for ACL reconstruction is further supported in a comprehensive narrative review published by Viswanthan et al. in January 2024 which compares the roles of PLT and HT autografts. The review concluded that PLT autografts demonstrate comparable functional outcomes and survival rates in comparison to alternative autograft options [25]. It also revealed that dimensions (including diameter and length) of harvested PLT autografts are relatively more consistent than HT autografts, with lower rates of complications and reduced risk of donor-site morbidity [25]. Nonetheless, several limitations for this review were highlighted, including a predominant reliance on non-clinical studies, clinical studies with relatively short follow-up periods, and less-reliable outcome evaluations [25]. This further underscores the imperative need for larger clinical trials with extended follow-up durations and the use of validated and uniform outcome measures, to conclusively delineate the role of PLT autografts in ACL reconstruction.

## Conclusions

The results of this SRMA including 683 patients from 6 RCTs suggest that the PLT autograft is a promising and non-inferior alternative to the HT autograft for ACL reconstruction, demonstrating equivalent early outcomes in patient-reported knee and ankle metrics, comparable graft diameters and fewer donor-site complications. Although these findings are based on level 1 RCT studies, it’s important to acknowledge the limitations present in the current literature. Therefore, while the results are promising, a degree of caution is advisable when considering their implications and further studies with larger sample sizes and longer follow-up would be beneficial.

## References

[1] Dallo I, Chahla J, Mitchell JJ, Pascual-Garrido C, Feagin JA, LaPrade RF (2017) Biologic approaches for the treatment of partial tears of the anterior cruciate ligament: a current concepts review. Orthop J Sports Med 5(1):2325967116681724. 10.1177/2325967116681724 28210653 10.1177/2325967116681724 PMC5298533

[2] van der List JP, Vermeijden HD, Sierevelt IN, Rademakers MV, Falke MLM, Helmerhorst GTT et al (2021) Repair versus reconstruction for proximal anterior cruciate ligament tears: a study protocol for a prospective multicenter randomized controlled trial. BMC Musculoskelet Disord 22:399. 10.1186/s12891-021-04280-y 33931067 10.1186/s12891-021-04280-y PMC8088019

[3] Arnold MP, Calcei JG, Vogel N, Magnussen RA, Clatworthy M, Spalding D et al (2021) ACL study group suvey reveals the evolution of anterior cruciate ligament reconstruction graft choice over the past three decades. Knee Surg Sports Traumatol Arthrosc 29:3871–3876. 10.1007/s00167-021-06443-9 33486558 10.1007/s00167-021-06443-9

[4] Galeazzi R (1934) La ricostruzione dei legamenti crociati del ginocchio Atti e memorie della Società Lombarda di Chirurgia. Milan

[5] D’Ambrosi R, Meena A, Arora ES, Attri M, Schäfer L, Migliorini F (2023) Reconstruction of the anterior cruciate ligament: a historical view. Ann Translat Med 11(10):364. 10.21037/atm-23-87 10.21037/atm-23-87 PMC10477645 37675316

[6] Zein AMN, Ali M, Zenhom Mahmoud A, Omran K (2017) Autogenous hamstring-bone graft preparation for anterior cruciate ligament reconstruction. Arthrosc Tech 6(4):e1253–e1262. 10.1016/j.eats.2017.04.011 29354425 10.1016/j.eats.2017.04.011 PMC5622011

[7] Kerimoğlu S, Aynaci O, Saraçoğlu M, Aydin H, Turhan AU (2008) Peroneus longus tendonu ile ön çapraz bağ rekonstrüksiyonu. Acta Orthop Traumatol Turc 42(1):38–43. 10.3944/aott.2008.038 18354276 10.3944/aott.2008.038

[8] Goncharov EN, Koval OA, Bezuglov EN, Vetoshkin AA, Goncharov NG, Encarnación Ramirez M et al (2023) Outcome of primary anterior cruciate ligament reconstruction with peroneus longus and bone-patellar tendon–bone autografts: a clinical comparative study. Surgeries 4(3):434–445. 10.3390/surgeries4030043 10.3390/surgeries4030043

[9] Zhao J, Huangfu X (2012) The biomechanical and clinical application of using the anterior half of the peroneus longus tendon as an autograft source. Am J Sports Med 40(3):662–671. 10.1177/0363546511428782 22174343 10.1177/0363546511428782

[10] Page MJ, McKenzie JE, Bossuyt PM, Boutron I, Hoffmann TC, Mulrow CD et al (2021) The PRISMA 2020 statement: an updated guideline for reporting systematic reviews. BMJ 372:n71. 10.1136/bmj.n71 33782057 10.1136/bmj.n71 PMC8005924

[11] Sterne JAC, Savović J, Page MJ, Elbers RG, Blencowe NS, Boutron I et al (2019) RoB 2: a revised tool for assessing risk of bias in randomised trials. BMJ 366:l4898. 10.1136/bmj.l4898 31462531 10.1136/bmj.l4898

[12] Luo D, Wan X, Liu J, Tong T (2018) Optimally estimating the sample mean from the sample size, median, mid-range, and/or mid-quartile range. Stat Methods Med Res 27(6):1785–1805. 10.1177/0962280216669183 27683581 10.1177/0962280216669183

[13] Agarwal A, Singh S, Singh A, Tewari P (2023) Comparison of functional outcomes of an anterior cruciate ligament (ACL) reconstruction using a peroneus longus graft as an alternative to the hamstring tendon graft. Cureus 15(4):e37273. 10.7759/cureus.37273 37168157 10.7759/cureus.37273 PMC10164842

[14] Bi M, Zhao C, Zhang S, Yao B, Hong Z, Bi Q (2018) All-inside single-bundle reconstruction of the anterior cruciate ligament with the anterior half of the peroneus longus tendon compared to the semitendinosus tendon: a two-year follow-up study. J Knee Surg 31(10):1022–1030. 10.1055/s-0038-1627466 29421837 10.1055/s-0038-1627466

[15] Saeed UB, Ramzan A, Anwar M, Tariq H, Tariq H, Yasin A et al (2023) Earlier return to sports, reduced donor-site morbidity with doubled peroneus longus versus quadrupled hamstring tendon autograft in ACL reconstruction. JB JS Open Access 8(4):e23.00051. 10.2106/JBJS.OA.23.00051 38058511 10.2106/JBJS.OA.23.00051 PMC10697627

[16] Shi FD, Hess DE, Zuo JZ, Liu SJ, Wang XC, Zhang Y et al (2019) Peroneus longus tendon autograft is a safe and effective alternative for anterior cruciate ligament reconstruction. J Knee Surg 32(8):804–811. 10.1055/s-0038-1669951 30206913 10.1055/s-0038-1669951

[17] Vijay C, Santosh MS, Avinash C, Adarsh T (2022) Is Peroneus longus autograft a better alternative to the hamstring autograft for anterior cruciate ligament reconstruction? A randomised control study. J Orthop Trauma Rehabilit 29(1):221049172210883. 10.1177/22104917221088335 10.1177/22104917221088335

[18] Waly AHT, Gawish HM (2022) Comparative study between peroneus longus, semitendinosus tendon, and quadriceps tendon graft for anterior cruciate ligament reconstruction: short-term results. Egypt Orthop J 57(2):109–121. 10.4103/EOJ.EOJ-57-109 10.4103/EOJ.EOJ-57-109

[19] de Sa D, Shanmugaraj A, Weidman M, Peterson DC, Simunovic N, Musahl V et al (2018) All-inside anterior cruciate ligament reconstruction—a systematic review of techniques, outcomes, and complications. J Knee Surg 31(9):895–904. 10.1055/s-0038-1627446 29528481 10.1055/s-0038-1627446

[20] Chan HY, Chen JY, Zainul-Abidin S, Ying H, Koo K, Rikhraj IS (2017) Minimal clinically important differences for American Orthopaedic Foot and Ankle Society score in hallux valgus surgery. Foot Ankle Int 38(5):551–557. 10.1177/1071100716688724 28193121 10.1177/1071100716688724

[21] Quinn M, Byrne RA, Albright JA, Testa E, Ahn B, Lemme N et al (2023) Peroneus longus tendon autograft may present a viable alternative for anterior cruciate ligament reconstruction: a systematic review. Arthrosc J Arthrosc Relat Surg 40(4):1366-1376.e1 10.1016/j.arthro.2023.10.016 37898307

[22] Shi W, Guo Z, Deng H, Shen Y, Shi L, Wu S et al (2020) Systematic evaluation of anterior cruciate ligament reconstruction with peroneus longus tendon and hamstring tendon. Chin J Tissue Eng Res 24(5):811–820. 10.3969/j.issn.2095-4344.1964 10.3969/j.issn.2095-4344.1964

[23] Zuo J, Shi F (2018) Clinical observation of the curative effects of arthroscopic peroneus longus tendon reconstruction in elderly patients with acute anterior cruciate ligament ruptures. Chin Gen Pract 21(30):3718–3722. 10.12114/j.issn.1007-9572.2018.00.037 10.12114/j.issn.1007-9572.2018.00.037

[24] Shair NA, Siddiq UAB, Tariq A, Khalid M, Mian MH (2022) Anterior cruciate ligament reconstruction with hamstring tendon autografts versus peroneus longus tendon autografts in isolated anterior cruciate ligament injury. Rawal Med J 47(2):362–366

[25] Viswanathan VK, Lyengar KP, Jain VK (2024) The role of peroneus longus (PL) autograft in the reconstruction of anterior cruciate ligament (ACL): a comprehensive narrative review. J Clin Orthop Trauma 49:102352. 10.1016/j.jcot.2024.102352 38356688 10.1016/j.jcot.2024.102352 PMC10862405

